# Gene Designer: a synthetic biology tool for constructing artificial DNA segments

**DOI:** 10.1186/1471-2105-7-285

**Published:** 2006-06-06

**Authors:** Alan Villalobos, Jon E Ness, Claes Gustafsson, Jeremy Minshull, Sridhar Govindarajan

**Affiliations:** 1 DNA 2.0, Inc. 1430 O'Brien Drive Suite E, Menlo Park, CA 94025, USA

## Abstract

**Background:**

Direct synthesis of genes is rapidly becoming the most efficient way to make functional genetic constructs and enables applications such as codon optimization, RNAi resistant genes and protein engineering. Here we introduce a software tool that drastically facilitates the design of synthetic genes.

**Results:**

Gene Designer is a stand-alone software for fast and easy design of synthetic DNA segments. Users can easily add, edit and combine genetic elements such as promoters, open reading frames and tags through an intuitive drag-and-drop graphic interface and a hierarchical DNA/Protein object map. Using advanced optimization algorithms, open reading frames within the DNA construct can readily be codon optimized for protein expression in any host organism. Gene Designer also includes features such as a real-time sliding calculator of oligonucleotide annealing temperatures, sequencing primer generator, tools for avoidance or inclusion of restriction sites, and options to maximize or minimize sequence identity to a reference.

**Conclusion:**

Gene Designer is an expandable Synthetic Biology workbench suitable for molecular biologists interested in the *de novo *creation of genetic constructs.

## Background

DNA, like any other form of information, can be both written and read. For DNA, reading is done by DNA sequencing and writing by gene synthesis. Most of molecular biology over the last decade has focused on reading and analyzing naturally occurring DNA sequences as revealed by massive worldwide sequencing efforts. In contrast, the emerging field of Synthetic Biology aims to write new genetic information, thereby creating designed non-natural genes, proteins, biological processes and organisms[1]. Gene synthesis was conceived as a means of gene acquisition in the 1970s and early 1980s[2,3], but was soon overtaken by cloning from libraries and later by PCR. More recently, protein and DNA sequences have become easier to obtain electronically through databases than physically from library clones. At the same time gene synthesis technology has matured. Direct synthesis of genes is rapidly becoming the most efficient way to make functional genetic constructs and enables applications such as codon optimization[4], making RNAi resistant genes[5] and protein engineering[6].

Synthetic Biology is the convergence of molecular biology and engineering principles that is underpinned by increasingly efficient technologies for creating full length genes, operons and even genomes *denovo *[7-9]. Codon optimization for heterologous protein expression has been shown to often drastically increase protein expression levels[4]. Central to such efforts is the ability to design the genetic constructs as easily as possible while considering multiple design parameters in parallel. For example, considerations such as codon bias use in the desired expression system, avoidance of mRNA secondary structures, degree of sequence identity to homologs and the presence or absence of specific restriction sites or motifs must all be considered simultaneously.

Current commercial sequence manipulation packages are typically very feature rich with graphic user interfaces and multiple integrated tools to allow for a seamless workflow. These commercial packages are primarily built to read and analyze sequence information, giving very little freedom to design and write new genetic information. On the other hand there are a plethora of freely available software that allow the user to simply codon optimize a sequence. These free tools are usually poor on gene design features, rely on a static web interface, are never updated, and have very limited flexibility. A representative selection of free codon optimization tools can be found in table 1 and also in reference[10]. These free codon optimization tools rarely use probabilistic algorithms, do not support features such as 'optimize close to' or 'far away from' a reference sequence, do not flag methylation sensitive restriction enzymes or capture manual editing in real time etc. These are all features that are incorporated in the codon optimization module of Gene Designer. Equally important, Gene Designer is built to integrate codon optimization with all the tools necessary to design, write and edit sequence information within one unifying user friendly interface. The Gene Designer software enables the quick, reliable and robust creation of new genetic information, a process essential for Synthetic Biology.

## Results

### Input and manipulation of data

Gene Designer is easy and intuitive to learn. It has a graphically rich molecular viewer for displaying and manipulating genetic constructs using simple drag-and-drop manipulations, coupled with a hierarchical data structure for storing, managing and accessing sequence objects. Gene Designer is a stand-alone secure software that provides an efficient integrated solution for gene design projects.

New sequence objects in Gene Designer can be entered as AA (amino acid sequence), DNA (nucleotide sequence object) or ORF (amino acid sequence linked to a nucleotide sequence). Each object can be imported directly in FASTA format or manually imported by cut-and-paste into a data entry window. Once loaded, each object can be displayed in icon, sequence or notes (annotation) view.

A set of commonly used genetic objects are provided in a tree structured Design Toolbox. The list includes prokaryotic and eukaryotic transcriptional and translational regulatory elements, purification and solubility tags, protease cleavage sites, secretion signals, restriction sites and recombinase cloning elements. The toolbox is not a complete and final list of genetic elements, but rather a convenient starting point for each user to assemble their own custom set of genetic objects. The software is built to enable the user to add and edit new custom objects and make notes associated with each object. These objects can be saved in the toolbox and can be shared between users. For detailed and up to date information of each existing building block, or to create new building blocks, we recommend the user searching NCBI databases and the World Wide Web.

The Icon View provides an immediate overview of the entire design project. Each genetic object is shown as a differently colored arrow indicating the orientation of the object. Objects can be moved in this view by drag-and-drop. This is particularly convenient when moving affinity tags from the N to the C-terminal of a protein, creating chimeric proteins and editing restriction sites at ends of a construct.

The Sequence View provides a detailed display of the nucleotide and/or amino acid sequences of each object below a single nucleic acid sequence corresponding to the entire construct. For AA objects, each amino acid (single letter code) is shown immediately above its corresponding codons. Codons are shown in descending order of their frequency in the corresponding codon usage table.

The Notes View provides a convenient way for the user to annotate the sequence elements for future reference. There is also a feature in the Notes View for reports on the entire project.

### Codon optimization

The genetic code uses 64 nucleotide triplets (codons) to encode 20 amino acids and stop. Each amino acid is encoded by on average 3 codons that are read during translation by tRNAs charged with the cognate amino acid. The degeneracy of the genetic code enables many alternative nucleotide sequences to encode the same protein. The frequencies with which different codons are used by different organisms and different types of genes vary significantly[11] and are correlated to the concentration of the corresponding tRNA population in the cell[12]. Rare codons are not only strongly associated with low levels of protein expression due to ribosome stalling and abortive translation[13], but also implicated in frameshift and amino acid misincorporation[14,15]. Codon usage has been identified as the single most important factor in prokaryotic gene expression[16].

The simplest way to design a DNA sequence from an amino acid sequence is to assign the most abundant codon to all instances of that amino acid in the sequence. Codon usage preference in a gene is often measured by Codon Adaptation Index (CAI score). The CAI score for such a construct is 1.0, *i.e*. in each case only the most abundant codon is used. This 'one amino acid – one codon' or 'CAI = 1.0' approach has several drawbacks. First, a strongly transcribed mRNA from such a gene will generate high codon concentrations for a subset of the tRNA populations, resulting in imbalanced tRNA pool, skewed codon usage pattern and increased translational error[17]. Heterologously expressed proteins may be produced at levels as high as 60% of total cell mass, making an imbalance tRNA pool a significant problem resulting in reduced growth due to tRNA depletion[18] and increased frameshift due to translational pausing at the ribosomal A-site[19]. Second, with no flexibility in codon selection, it is impossible to avoid repetitive elements and mRNA secondary structures in the gene. Severe repetitive elements can affect the genetic stability of a gene and may lead to excision through recombination. Third, it is often desirable to incorporate or exclude sequence elements such as restriction sites from the sequence to facilitate subsequent manipulations. These modifications are impossible to accommodate if the codon usage is rigidly fixed. Fourth, in the literature there are many and sometimes conflicting data suggesting sequence elements that decrease protein expression levels. Such elements can not be avoided if the codon usage is fixed. Gene Designer users who wish to use the CAI = 1 optimization approach can either increase the threshold for codons used or use a modified codon usage table.

In contrast to the 'CAI = 1.0' method, Gene Designer optimizes genes for expression by using a codon usage table in which each codon is given a probability score based on the frequency distribution of the codons in the genome normalized for every amino acid. The codon usage tables for 25 common protein expression hosts are included with the download, and new codon usage tables can be imported from the Codon Usage Database [20] or manually edited as required. The codon usage table created by one user is automatically imported when another user shares the project. For *E. coli *expression we recommend the user to use the EColi_CII table that is derived from a collection of highly expressed *E. coli *genes[21]. Candidate sequences are generated *in silico *using a Monte Carlo algorithm by selecting codons based on the probabilities obtained from the codon usage table, with codons below the threshold value (default is 10%) excluded from consideration. Each designed sequence is then passed through subsequent iterations to ensure a match with additional design criteria such as filtering out mRNA secondary structures and DNA repeats, eliminating or incorporating restriction sites and avoiding methylation sites that overlap methylation sensitive restriction sites[4]. A pseudo code for the algorithm in Gene Designer can be found in appendix A.

Motifs such as internal Shine-Dalgarno sequences have been shown to decrease gene expression[22]. Gene Designer allows the user to filter out Shine-Dalgarno sequences, splice donor and acceptor sequences as well as any other sequence motif defined by the user. The user can also maximize or minimize the similarity of the designed sequence to a reference sequence, for example to make RNAi-resistant genes[5] or to maximize the probability of recombination between two variants. Since the algorithm is a Monte Carlo based algorithm where each codon choice is an independent probabilistic event, the software can iterate the optimization each time finding a new and equally good solution.

Gene Designer does not utilize advanced RNA folding calculation software such as the popular mFold[23] as these types of software are designed to calculate RNA secondary structures for naked RNA. The translated mRNA within an ORF is in fact densely covered by ribosomes. Chemical footprinting of mRNA-ribosome complexes show that up to 20 codons (60 bases) are covered by a single translating ribosome[24], and the ribosomes are translating at ~18 codons (54 bp)/sec with one ribosome initiating translation every ~2 second[25] leaving only ~50 mRNA bases available between translating ribosomes for folding an mRNA secondary structure. During translation, a stem-loop structure in the coding part of the mRNA does not hinder the progress of the translational machinery, and actively translating ribosomes can break up such structures, either by the energy driven translation process itself or by the support of RNA helicases [26-28].

Gene Designer filters out (or flags, if it can not be avoided) any mRNA structure with double-stranded RNA stem of 12 bp or more. This feature is included because it is very often requested by users and also because it ensures that oligonucleotides used in the gene synthesis process will not predominantly self-anneal during gene assembly.

The codons immediately 3' of the initiation ATG codon have a strong influence on gene expression[22,29-31]. Accordingly, the codon optimization module in Gene Designer gives the user the option to treat the 5' end of the ORF separately. The default is conservatively set to include the first 15 codons of the ORF as 5' end, but can be changed as needed. Gene Designer will filter out NGG codons in the 5' region[32] and predominantly use A/T in the wobble position[33,34]. The 5' end is also set to filter out repeats of 8 bases or more and filter out mRNA secondary structures of 8 bp or more.

The local context of a codon can influence the protein expression levels. Back in the early 1980s it was shown that the efficiency of the UAG stop codon in *E. coli *is typically decreased in the presence of a 3' adenine and increased in the presence of a 3' cytidine[35,36]. Since then, a multitude of experimentally validated codon contexts have been shown to affect ribosomal frameshift, missense and nonsense incorporations and translational efficiency [37-40]. Gene Designer avoids known codon context issues by omitting the use of rare codons and filtering out runs of C's and G's. We also recommend the addition of two stop codons at the end of an ORF to ensure proper translational termination.

Aside from the experimentally validated cases of codon context effect on protein expression levels, there are several publications where in which codon context effects have been proposed based on *in silico *analysis of genomes [41-43]. The absence or low level of certain codon contexts in the analysis of entire genomes does not necessarily reflect that the identified sequences affect protein expression of a recombinant gene when grown in rich media, but more likely is a consequence of other evolutionary pressures such as facilitating DNA replication, mutational bias, expression during starvation, intrinsic metabolic regulation etc.. [44] In at least one case[45], the predicted codon pair bias effect on protein expression could not be experimentally validated[46]. The current version of Gene Designer only includes pre-set sequence constraints that have been experimentally validated. The individual user may add to these any sequence elements they wish to eliminate.

### Other design features

Any object can be split into two or more daughters by selecting a part of the sequence and using the Split function. Users can thus easily divide proteins into domains for easy drag-and-drop construction of chimeras or gene variants. Objects can also be linked within and between projects; changes in linked objects then propagate throughout all open projects. All changes, such as editing an object's sequence, changing codon table or codon threshold are incorporated into the final sequence in real time.

The Gene Designer can also be used to design oligonucleotides. To assist with this, a real-time Tm calculator can be positioned in the Sequence View and dragged until a preferred location, length and melting temperature is found. The DNA melting temperature calculation is performed using the nearest neighbor method[47,48]. The software can also design sequencing primers for a specified region or spanning the entire construct through an integrated 'Actions' module.

Once a sequence has been designed, sequences can be saved with all the graphical elements and captured relationships as Gene Design files (.gd suffix), saved as a graphic image (.jpeg) or as plain text (.txt). Reports can be generated that contain the complete nucleotide sequence, the nucleotide sequence of each object, notes, translation map of each object, a restriction site summary, codon usage frequencies and GC content. Finally, by clicking the 'Get quotation' or 'Order gene' icon, the designed synthetic DNA fragment can be priced or placed in the gene synthesis pipeline of DNA 2.0.

## Conclusion

Gene Designer provides an easily accessible means of designing synthetic genes, operons and other genetic constructs denovo. The user can combine and modify pre-defined and custom genetic building blocks directly through a user friendly drag-and-drop interface. All manipulations needed for gene design are integrated and immediately accessible under one interface.

The authors are using and have been using Gene Designer daily over the last year. Several thousand genes have now been designed using only this software. The savings in time, increased convenience and reliability of Gene Designer compared to other commercial and freeware tools has dramatically improved our efficiency and ensure a robust pipeline for sequence information handling. Furthermore, applications such as creating RNAi resistant genes[5] could only be enabled using the Gene Designer software.

Please contact the authors to suggest features to include in upcoming Gene Designer releases.

## Availability and requirements

Gene Designer is freely available for download from the 'Tools' menu at . Both Mac and PC versions are available. The software is provided "as is" with no guarantee or warranty of any kind for non-commercial use. Please see the download licensing agreement for further licensing details and restrictions on commercial use.

## Authors' contributions

AV developed the software, implemented the algorithms and participated in designing the interface. JN and JM conceived the software and features to be included and participated in designing the interface and testing the software. JM also wrote the Help section of the software. CG wrote the manuscript and participated in defining the scope of the software and testing it. SG lead the project, participated in developing, defining the scope as well as features of the software. All authors read and approved the final manuscript.

## Appendix A. Pseudo-code for codon optimization in Gene Designer

**FOR EACH **A.A. sequence

   **FOR EACH **codon in sequence

      Select a codon randomly from the probability distribution. †

**FOR EACH **A.A. sequence that needs homologue (aiming/avoidance)

   Prepare homologue alignment matrix.

   Pre select codons that are (closest to/furthest from) homologue sequence.

   **IF **homologue dna contains unwanted restriction sites or other unwanted sequences **THEN**

      Ask/warn user and eliminate if necessary.

Create a Ukkonen Suffix Tree of the entire construct concatenated with its reverse compliment.

*H *= homologue score for all A.A. sequences that require it.

*R *= number of repeats over given threshold.

*M *= size of largest repeat.

**WHILE ***R *> 0 **DO **‡

   Change a codon in the largest repeat region based on the probability distribution. †

   *H*_*new *_= homologue score after change.

   *R*_*new *_= number of repeats after change.

   *M*_*new *_= size of largest repeat after change.

   **IF ***H*_*new*_≥ *H ***AND (***R *<*R*_*new *_**OR ***M *<*M*_*new*_**) THEN**

      Accept change.

      *H *= *H*_*new*_

      *R *= *R*_*new*_

      *M *= *M*_*new*_

**FOR EACH **A.A. sequence that requires 5' translation optimization

   Create a Ukkonen Suffix Tree of the 5' end concatenated with its reverse compliment.

   Find hairpins in 5' end.

   *GC*_*goal *_= CG ratio wanted × 3 × number of codons being considered in 5' end.

   *H *= homologue score for the 5' end.

   *R *= number of hairpins.

   *GC *= total number of G's and C's in 5' end.

   **WHILE ***R *> 0 **OR ***GC *> *GC*_*goal *_**DO **‡

      Change a random codon in 5'end based on the probability distribution. †

      *H*_*new *_= homologue score after change.

      *R*_*new *_= number of hairpins after change.

      *GC*_*new *_= number of G's and C's after change.

      **IF***H*_*new*_≥ *H *AND **(***R*_*new *_<*R ***OR (***R*_*new *_= *R ***AND ***GC*_*new *_<*GC***)) THEN**

         Accept change.

         *H = H*_*new*_

         *R = R*_*new*_

         *GC = GC*_*new*_

**FOR EACH **restriction enzyme that needs to be checked for methylation

   Find methylated sites.

   **WHILE **still methylated **DO **‡

      Change a codon in the site based on the probability distribution. †

**FOR EACH **restriction enzyme that needs to be avoided.

   Find restriction sites.

   **WHILE **restriction site still exists **DO **‡

      Change a codon in the site based on the probability distribution. †

† Based on a given precompiled codon bias table.

‡ This can go on forever, must be stopped artificially after a given number of iterations

## Figures and Tables

**Figure 1:**
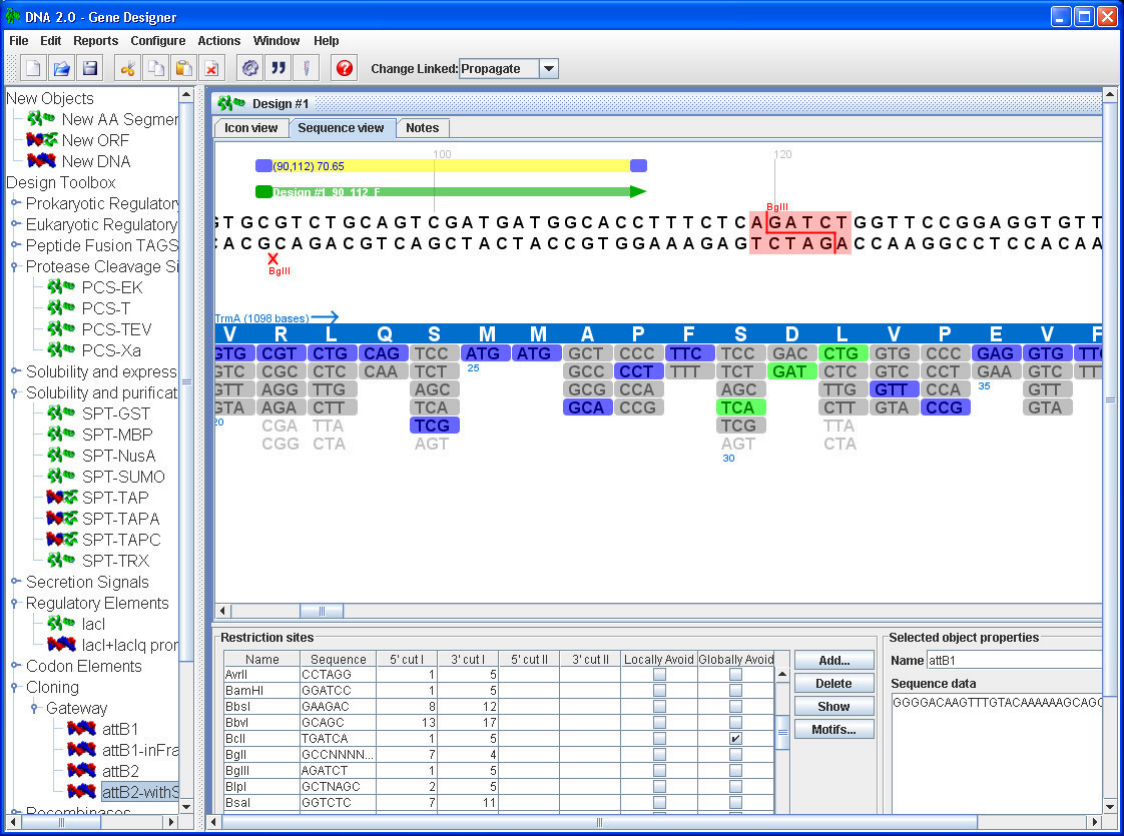
**Graphic user interface of Gene Designer**. Section of the Gene Designer environment in Sequence view (top) with restriction sites and object properties windows (bottom). Tm calculator and restriction-site denoted in Sequence view.

**Table 1:** Public codon optimization softwares.

**Name**	**Features**	**Comment**	**Reference**
	RE	Macintosh	[49]
GMAP	RE		[50]
CalcGene	CUT		[51]
COD OP	CUT, RE, DO	Unix perl	[52]
DNA Works	CUT, RE, DO	Web browser	[53]
Codon Optimizer	CUT, RE		[54]
Prot2DNA	CUT, RE, mSeq, Rep	Precursor to Gene Designer	[4]
Gene2Oligo	DO	Web browser	[55]
UpGene	CUT, DO		[56]
GeMS	CUT, RE, mSeq, DO	Web browser	[57]
JCat	CUT, RE	Web browser	[58]
Synthetic Gene Designer	CUT, RE, DO	Web browser	[59]

Software with features similar to the codon optimization module of Gene Designer. **CUT **indicates that one or more codon usage tables are referenced during reverse translation of the protein sequence. **RE **indicates that restriction endonuclease sites can be identified and silently removed and/or inserted. **Rep **indicates that repeats and/or inverted repeats can be silently removed. **mSeq **indicates that mRNA secondary structures can be silently removed. **DO **identifies software that includes a module for the design of oligonucleotides for gene synthesis
